# Prevalence of Glucose-6-Phosphate Dehydrogenase (G6PD) Deficiency and Risk of Hyperbilirubinemia Among Newborns: A Tertiary Center Experience from Western Saudi Arabia

**DOI:** 10.3390/pediatric18020059

**Published:** 2026-04-15

**Authors:** Rogaya AlShugair, Mansour Al-Qurashi, Ahmad Mustafa, Mohammad Y. Alhindi, Abrar Ahmed, Hend AlNajjar, Mona AlDabbagh, Ashraf Sahafi, Hashim Almarzouki, Nabila A. AlRashdi, Eman A. AlThobaiti, Syed Sameer Aga

**Affiliations:** 1 Department of Neonatal Intensive Care, King Abdullah Specialty Children’s Hospital, Jeddah 21423, Saudi Arabia; rogaya1992@gmail.com (R.A.); alhindimy1@yahoo.com (M.Y.A.); al_abrar_way333@hotmail.com (A.A.); dabbaghm@mngha.med.sa (M.A.); ahafias@mngha.med.sa (A.S.); rashdina@mngha.med.sa (N.A.A.); althobitiim@mngha.med.sa (E.A.A.); 2 College of Medicine, King Saud bin Abdulaziz University for Health Sciences, Jeddah 21423, Saudi Arabia; marzoukih@mngha.med.sa (H.A.); agas@ksau-hs.edu.sa (S.S.A.); 3 King Abdullah International Medical Research Center (KAIMRC), Jeddah 21423, Saudi Arabia; najjarh@ksau-hs.edu.sa; 4 College of Nursing, King Saud bin Abdulaziz University for Health Sciences, Jeddah 21423, Saudi Arabia; 5 Department of Pediatric Ophthalmology, King Abdullah Specialty Children’s Hospital, Jeddah 21423, Saudi Arabia

**Keywords:** G6PD deficiency, neonatal jaundice, hyperbilirubinemia, newborn screening, phototherapy, late preterm infants, Saudi Arabia

## Abstract

**Background:** Glucose-6-phosphate dehydrogenase (G6PD) deficiency is among the most common inherited enzymatic disorders worldwide and is an important risk factor for neonatal hyperbilirubinemia. Regional data from Western Saudi Arabia based on universal newborn screening remain limited. **Objectives:** To determine the prevalence of G6PD deficiency among newborns delivered at a tertiary center in Jeddah, Saudi Arabia, and to evaluate its association with clinically relevant outcomes, including early-onset jaundice (<24 h), need for phototherapy, admission for hyperbilirubinemia management, and readmission after discharge. **Methods:** We conducted a retrospective cohort study at King Abdulaziz Medical City, Western Region, Jeddah, Saudi Arabia, between January 2020 and May 2025. Cord blood samples from live-born infants were screened using a qualitative fluorescent spot test. Demographic variables (sex, gestational age, birth weight) and jaundice-related outcomes were extracted from the electronic medical record. Categorical variables were compared using chi-square testing, with *p* < 0.05 considered statistically significant. **Results**: Among 14,964 screened newborns, 489 were identified as G6PD deficient, yielding a prevalence of 3.3%. Prevalence was higher in males than in females (5.6% vs. 0.9%). Among the G6PD-deficient infants, early-onset jaundice occurred in 17.2%, phototherapy was required in 36.0%, and 16.5% were admitted for hyperbilirubinemia management. Readmission for worsening jaundice requiring phototherapy occurred in 11.0%, and no exchange transfusions were required. Compared with term infants, late preterm infants had higher rates of early-onset jaundice (11/49, 22.4% vs. 73/440, 16.6%) and phototherapy use (22/49, 45.0% vs. 154/440, 35.0%) (*p* < 0.01). **Conclusions:** G6PD deficiency was identified in a substantial proportion of newborns in this large screened cohort and was associated with clinically significant jaundice-related outcomes, particularly among late preterm infants. These findings underscore the importance of universal screening and structured postnatal follow-up to reduce the risk of severe hyperbilirubinemia and its complications. Early identification of G6PD-deficient infants should be accompanied by careful bilirubin monitoring, clear discharge planning, and timely post-discharge follow-up, especially for those born late preterm.

## 1. Introduction

Glucose-6-phosphate dehydrogenase (G6PD) deficiency is the most common inherited enzymatic disorder of red blood cells and affects hundreds of millions of individuals globally, with an estimated neonatal prevalence of 4.9% and significant geographic and demographic variations [1,2,3,4]. It is inherited in an X-linked pattern, resulting in a higher prevalence among males and variable phenotypic expression in females due to lyonization.

G6PD is a key enzyme in the hexose monophosphate (pentose phosphate) pathway and is essential for generating nicotinamide adenine dinucleotide phosphate (NADPH). NADPH maintains glutathione in its reduced form and protects erythrocytes and other tissues from oxidative stress. G6PD deficiency increases susceptibility to oxidative injury and hemolysis, particularly when exposed to triggers such as infection, certain medications, and fava beans. In neonates, G6PD deficiency is strongly associated with an increased risk of severe hyperbilirubinemia, which may present early, progress rapidly, and—if unrecognized—result in acute bilirubin encephalopathy or kernicterus [5,6,7].

The prevalence of G6PD deficiency varies widely across geographic regions and ethnic groups, with higher rates in malaria-endemic areas, including parts of Africa, Asia, and the Middle East [8,9,10,11,12,13,14,15,16,17]. In Saudi Arabia, prevalence is reported to vary by region and population characteristics, ranging from approximately 2% in lower-prevalence regions to 15–30% in high-prevalence areas, particularly in the Eastern Province [18,19,20,21,22,23,24]. However, data from Western Saudi Arabia remain comparatively limited. Importantly, the clinical impact of G6PD deficiency extends beyond prevalence; early neonatal outcomes such as early-onset jaundice, the need for phototherapy, and admission to higher levels of care have important implications for patient safety and resource utilization [5,11,12,13,14].

Late preterm infants (34 to <37 weeks’ gestation) constitute a clinically important subgroup, as physiological immaturity of hepatic bilirubin conjugation and increased enterohepatic circulation may amplify the risk of significant hyperbilirubinemia. When late preterm status coexists with G6PD deficiency, the risk of clinically significant hyperbilirubinemia may be further increased [13,25], yet this subgroup is frequently combined with term infants in routine reporting.

Accordingly, we aimed to determine the prevalence of G6PD deficiency among newborns delivered at a tertiary center in Jeddah using universal cord blood screening and to evaluate associations with early-onset jaundice, need for phototherapy, admission for further hyperbilirubinemia management, and readmission after hospital discharge. We also examined whether gestational age category (late preterm vs. term) and sex were associated with differential risk among G6PD-deficient infants.

## 2. Methods

### 2.1. Study Design and Setting

This was a retrospective cohort study conducted at King Abdulaziz Medical City—Western Region (KAMC-J), Ministry of National Guard Health Affairs, Jeddah, Saudi Arabia. The study period spanned from January 2020 to May 2025. The delivery unit, postnatal services, and neonatal intensive care unit (NICU) follow a universal newborn screening policy for G6PD deficiency using cord blood at the time of delivery.

### 2.2. Ethics Approval

The study was approved by the Institutional Review Board of King Abdullah International Medical Research Center (KAIMRC) (IRB No: NRJ24/028/11).

### 2.3. Study Population

All live-born neonates delivered during the study period who underwent routine cord blood screening for G6PD deficiency were included in the analysis. Newborns without a documented screening result were excluded. The screened cohort represented approximately 99.5% of live births delivered at the institution during the study period.

### 2.4. G6PD Screening Method

Cord blood samples obtained at delivery were screened for G6PD deficiency using a qualitative fluorescent spot test according to institutional laboratory standards. When results were borderline or equivocal, confirmatory testing using quantitative enzyme assay and/or repeat testing was performed according to institutional practice. Infants were classified according to the final documented laboratory result. Infants were classified as G6PD-deficient or normal based on the screening result. As a qualitative test, this approach may have limited sensitivity for mild deficiency and for heterozygous females with intermediate enzyme activity.

### 2.5. Variables and Outcomes

Bilirubin assessment and management during the study period followed institutional neonatal jaundice protocols based on the American Academy of Pediatrics (AAP) guidelines. The 2004 AAP recommendations were initially applied, with a later transition to the updated 2022 AAP guideline after its adoption. Bilirubin evaluation was performed using transcutaneous screening and/or serum bilirubin measurement as clinically indicated, and decisions regarding phototherapy and admission were guided by gestational age, postnatal age, bilirubin level, and clinical risk factors.

Demographic variables included sex, gestational age, and birth weight. Gestational age was categorized as late preterm (34 to <37 weeks) or term (≥37 weeks). Birth weight was categorized as low birth weight (<2500 g) or appropriate birth weight (≥2500 g). Jaundice-related clinical outcomes among G6PD-deficient infants included (1) early-onset jaundice, defined as clinically visible jaundice within the first 24 h of life, confirmed by transcutaneous and/or serum bilirubin measurement; (2) requirement for phototherapy during the hospital stay before discharge; (3) admission for further management of hyperbilirubinemia; (4) readmission within 5–14 days after discharge due to worsening hyperbilirubinemia requiring phototherapy; and (5) need for exchange transfusion.

### 2.6. Data Sources and Extraction

Data were extracted from the electronic medical record system (BestCare) by chart review, including screening results and clinical outcomes. Extracted data were de-identified and stored on secure institutional systems.

### 2.7. Statistical Analysis

Analyses were performed using SPSS software (version 27.0). Categorical variables are presented as frequencies and percentages. Comparisons between late preterm and term infants within the G6PD-deficient group were conducted using the chi-square test. A two-sided *p* value < 0.05 was considered statistically significant.

## 3. Results

**Figure 1:**
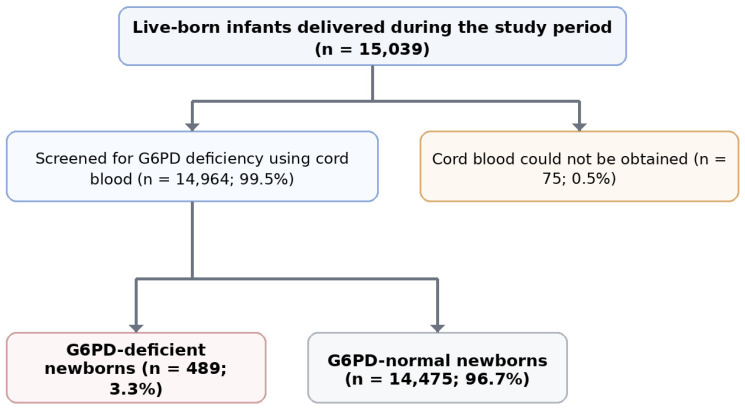
Study flow diagram of screened neonates and the G6PD-deficient cohort.

A total of 14,964 newborns were screened during the study period. G6PD deficiency was identified in 489 infants, yielding an overall prevalence of 3.3%. Prevalence was higher among males than females, consistent with X-linked inheritance; 424 of 7560 males (5.6%) were G6PD deficient compared with 65 of 7404 females (0.9%) (*p* < 0.001) (Figure 1, Table 1).

Gestational age distribution among screened newborns included 1490 late preterm infants (10.0%) and 13,474 term infants (90.0%). The prevalence of G6PD deficiency was similar across gestational age categories (3.3% in both late preterm and term infants). In the overall screened cohort, mean (±SD) gestational age was 38.2 ± 1.5 weeks, and mean (±SD) birth weight was 3050 ± 520 g (Table 1).

Among G6PD-deficient infants (n = 489), early-onset jaundice within the first 24 h occurred in 84 (17.2%), phototherapy was required in 176 (36.0%), and 81 (16.5%) were admitted for further management of hyperbilirubinemia (Table 2). Readmission within 5–14 days after discharge for worsening hyperbilirubinemia requiring phototherapy occurred in 54 (11.0%). None of the affected infants required an exchange transfusion.

**Figure 2:**
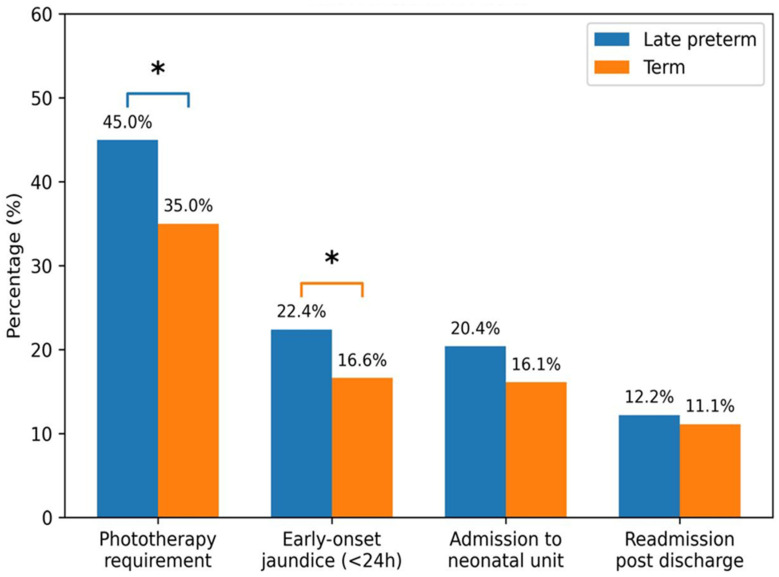
Clinical outcomes in G6PD-deficient infants: late preterm vs. term. The asterisk represent the significant differences (*p* < 0.05) in variables.

Late preterm infants with G6PD deficiency (n = 49) had higher rates of jaundice-related outcomes than term infants with G6PD deficiency (n = 440). Rates of early-onset jaundice were 22.4% in late preterm versus 16.6% in term infants (*p* < 0.01), and phototherapy was required in 45.0% versus 35.0%, respectively (*p* < 0.01). Admission rates for hyperbilirubinemia management were higher in late preterm infants (20.4% vs. 16.1%) but did not reach statistical significance (*p* = 0.12). Readmission rates were similar (12.2% vs. 11.1%; *p* = 0.92). (Table 2, Figure 2).

A sex-specific comparison demonstrated a substantially higher prevalence of G6PD deficiency among males than females (5.6% vs. 0.9%, *p* < 0.001), consistent with the X-linked inheritance pattern. Among affected infants, jaundice-related outcomes were higher among males; however, none of these differences reached statistical significance. Phototherapy was required in 37.5% of male infants compared with 26.2% of females. Similarly, admission for hyperbilirubinemia management (17.2% vs. 12.3%) and early-onset jaundice within the first 24 h (17.5% vs. 15.4%) were more frequent among males. However, none of these outcome differences reached statistical significance (Table 3).

## 4. Discussion

In this large single-center cohort spanning over 65 months of universal newborn screening, G6PD deficiency was identified in 3.3% of screened newborns. This prevalence is consistent with the broad range of estimates reported across Saudi Arabia [18,19,20,21,22,23,24,25,26], where regional variation has been described.

As expected for an X-linked disorder, G6PD deficiency was more prevalent among male infants; however, 14% of affected infants were female. Although jaundice-related outcomes were higher in males, these differences were not statistically significant, likely reflecting the smaller number of affected females. Importantly, available evidence suggests that the severity of neonatal hyperbilirubinemia is determined primarily by residual enzyme activity rather than sex alone. Therefore, both male and female infants with confirmed G6PD deficiency require similar levels of jaundice surveillance and follow-up [5,18].

In our cohort, more than one-third of G6PD-deficient infants required phototherapy, and approximately one in six developed early-onset jaundice within the first 24 h. A substantial proportion required admission for hyperbilirubinemia management, and 11% were readmitted shortly after discharge due to worsening jaundice requiring phototherapy. Notably, no exchange transfusions were required in this cohort, which may reflect early identification through universal screening, standardized bilirubin monitoring practices, and timely access to phototherapy. Neonatal hyperbilirubinemia in G6PD deficiency is often not primarily hemolytic; rather, impaired bilirubin conjugation and hepatic immaturity are believed to play major roles [5,6,7].

A key finding of this study is the heightened vulnerability of late preterm infants with G6PD deficiency. Compared with term infants, late preterm infants had significantly higher rates of early-onset jaundice and phototherapy requirements. These findings are biologically plausible and clinically important [6,7,15]. When combined with G6PD deficiency—through increased oxidative susceptibility and altered bilirubin handling—the risk of clinically significant hyperbilirubinemia is amplified [15,18].

These findings support a risk-stratified approach to bilirubin surveillance in newborns. Universal screening enables early identification of G6PD-deficient infants at birth, but screening alone is insufficient unless coupled with structured clinical pathways. For affected infants, particularly those born late preterm, clinical pathways should include early bilirubin measurement, closer inpatient monitoring for rapid rises in bilirubin, explicit discharge planning, and timely outpatient follow-up within the first postnatal week. The observed 11% readmission rate in our cohort highlights the need for proactive follow-up after discharge.

Comparison with published local and international literature suggests that the prevalence and clinical burden of G6PD deficiency vary by region, population genetics, and screening and management protocols. Differences may also reflect the spectrum of enzymatic activity (including heterozygous females), assay methodology (qualitative vs. quantitative), and thresholds for the use of phototherapy. Importantly, the rate of phototherapy use and early-onset jaundice in our cohort demonstrate the clinical relevance of G6PD deficiency even in settings with established bilirubin surveillance.

From a public health perspective, integrating G6PD screening into universal newborn screening programs offers a practical strategy to reduce severe hyperbilirubinemia and prevent kernicterus [4,15,18], particularly in regions with non-trivial prevalence. Moreover, family education regarding the avoidance of oxidative triggers later in infancy and childhood remains essential, although neonatal jaundice may occur even without clear external triggers. This study also provides contemporary data from Western Saudi Arabia, a region for which published neonatal screening-based prevalence data are less available than in other provinces. Our large sample size and consistent institutional screening approach strengthen confidence in the prevalence estimate and characterization of clinically relevant outcomes.

### Strengths and Limitations

Strengths of this study include the large screened cohort, near-universal screening coverage at the institution, and the use of clinically relevant outcomes that reflect patient burden and resource utilization.

Several limitations should be considered. First, this is a single-center retrospective study; therefore, findings may not be fully generalizable to other regions or institutions with different population genetics, screening strategies, and clinical pathways. Second, screening relied on a qualitative fluorescent spot test performed on cord blood rather than a quantitative enzyme activity assay. Qualitative testing may underestimate mild or intermediate deficiency, particularly among heterozygous females, potentially leading to misclassification and underestimation of prevalence or outcome associations. Third, as a retrospective analysis, important unmeasured confounders (e.g., feeding type, excessive postnatal weight loss, ABO incompatibility, and other contributors, including the presence of cephalohematoma and sepsis) may have influenced jaundice outcomes, and treatment thresholds may have varied over time based on evolving clinical practice guidelines.

## 5. Conclusions

G6PD deficiency affected 3.3% of newborns delivered at our center in Western Saudi Arabia and was associated with substantial jaundice-related morbidity. More than one-third of affected infants required phototherapy, and late preterm infants experienced significantly higher rates of early-onset jaundice and phototherapy use compared with term infants. Universal newborn screening, coupled with targeted bilirubin surveillance and structured post-discharge follow-up—especially for late preterm infants—may reduce the risk of severe hyperbilirubinemia and its sequelae. Future multicenter studies using quantitative enzyme assays and genetic characterization are needed to refine risk stratification and optimize screening and management protocols in Saudi Arabia.

## Figures and Tables

**Table 1:** Study population demographics and prevalence of G6PD deficiency (2020–2025).

Characteristic/ Variable	Total Screened Newborns (n = 14,964)	G6PD-Deficient Newborns (n = 489)
**Sex**		
- Male	7560 (50.5%)	424 (86.7%)
- Female	7404 (49.5%)	65 (13.3%)
**Gestational age category** (Mean 38.2 ± 1.5)		
- Late preterm (34–<37 wk)	1490 (10.0%)	49 (10.1%)
- Term (≥37 weeks)	13,474 (90.0%)	440 (89.9%)
**Birth weight category** (Mean 3050 ± 520)		
- <2500 (g)	1990 (13.3%)	63 (12.9%)
- ≥2500 (g)	12,974 (86.7%)	426 (87.1%)
Percentages are the representation of each sub-category of the variables.		

**Table 2:** Clinical outcomes among G6PD-deficient newborn infants (n = 489). Total cohort vs. late preterm vs. term infants.

Outcome	Total (n = 489)	Late Preterm (n = 49)	Term (n = 440)	*p* Value
**✓ Early-onset jaundice (<24 h)**	84 (17.2%)	11 (22.4%)	73 (16.6%)	**<0.01**
**✓ Required phototherapy**	176 (36.0%)	22 (45.0%)	154 (35.0%)	**<0.01**
**✓ Admission for hyperbilirubinemia management**	81 (16.5%)	10 (20.4%)	71 (16.1%)	0.12
**✓ Readmission within 5–14 days**	54 (11.0%)	6 (12.2%)	48 (11.1%)	0.92
**✓ Exchange transfusion**	0 (0%)	0 (0%)	0 (0%)	—

**Table 3:** Sex-specific characteristics and outcomes among G6PD-deficient infants.

Outcome/Variable	Male Infants (n = 424)	Female Infants (n = 65)	*p* Value
**Sex-specific incidence**	5.6%	0.9%	**<0.001**
**Mean gestational age (weeks) ± SD**	38.7 ± 1.9	38.5 ± 2.2	0.10
**Mean birth weight (g) ± SD**	3000 ± 510	2880 ± 500	0.85
**Phototherapy for jaundice**	159 (37.5%)	17 (26.2%)	0.08
**Admission for hyperbilirubinemia management**	73 (17.2%)	8 (12.3%)	0.32
**Jaundice within first 24 h**	74 (17.5%)	10 (15.4%)	0.68

## Data Availability

De-identified data underlying this study may be made available by the corresponding author upon reasonable request and subject to institutional policies and required approvals.
